# The Association between Adiposity, Mental Well-Being, and Quality of Life in Extreme Obesity

**DOI:** 10.1371/journal.pone.0092859

**Published:** 2014-03-26

**Authors:** Alison C. Jagielski, Adrian Brown, Marzieh Hosseini-Araghi, G. Neil Thomas, Shahrad Taheri

**Affiliations:** 1 Birmingham and Black Country NIHR CLAHRC, Birmingham, United Kingdom; 2 Specialist Weight Management Services, Heart of England NHS Foundation Trust, Birmingham, United Kingdom; 3 Public Health, Epidemiology and Biostatistics, University of Birmingham, United Kingdom; 4 Institute of Public Health, Social and Preventive Medicine, Mannheim Medical Faculty, Heidelberg University, Mannheim, Germany; 5 Department of Medicine, Weill Cornell Medical College, New York, New York, United States of America, and Doha, Qatar; 6 Department of Medicine, King’s College London, London, United Kingdom; University of Florida, United States of America

## Abstract

**Objectives:**

To explore the cross-sectional association between adiposity, mental well-being, and quality of life in extreme obese individuals entering a UK specialist weight management service prior to treatment commencement.

**Methods:**

The sample comprised 263 extreme obese individuals who were referred to the service as a result of having a body mass index (BMI) ≥40 kg/m^2^ or ≥35 kg/m^2^ with a co-morbid health condition. In a retrospective analysis, routinely collected baseline clinical examination data and self-report questionnaires (Impact of Weight on Quality of Life: IWQOL-Lite, EQ5D-3L, and Hospital Anxiety and Depression Scale: HADS) were analysed to examine the cross-sectional association between adiposity and quality of life.

**Results:**

The sample was predominantly female (74.8%) with mean BMI 47.0±7.9 kg/m^2^. Increasing adiposity was significantly negatively associated with quality of life, with an increase of 1 BMI unit associated with decreases of 1.93 in physical function (95% CI −2.86 − −1.00, p<0.001), 1.62 in self-esteem (95% CI −2.67 − −0.57, p<0.05), 2.69 in public distress (95% CI −3.75 − −1.62, p<0.001), 1.33 in work (95% CI −2.63 − −0.02, p<0.05), and 1.79 in total IWQOL-Lite scores (95% CI −2.65 − −0.93, p<0.001). Adiposity was associated with significantly increased risk of problems in mobility (OR = 3.44, 95% CI 1.47−8.05), and performing usual activities (OR = 2.45, 95% CI 1.10−5.46) in highest relative to lowest BMI tertile. The prevalence of experience of symptoms of anxiety (70.3%) and depression (66.2%) as measured by HADS was consistently high.

**Conclusions:**

We identified a high prevalence of psychological co-morbidity, including widespread experience of symptoms of anxiety and depressive disorders and reduced quality of life among these extreme obese individuals seeking weight management treatment. Clinical implications include the need for the incorporation of strategies to improve mental well-being into multi-disciplinary weight management interventions.

## Introduction

The prevalence of obesity among adults, and in particular extreme obesity, has risen rapidly over previous decades [1]. Current levels of extreme obesity (BMI ≥40 kg/m^2^) in the US have increased 70% over the last decade between 2000 and 2010, with the prevalence rate reaching 6.6% [2], whilst current UK prevalence rates of extreme obesity are 3% for females and 2% for males [3]. Indeed, recent estimates predict the rate of extreme obesity to reach 5% in the UK by 2033 and 9% in the US by 2030 [4]. The physical co-morbidities of extreme obesity are well documented and research suggests that there are also substantial negative impacts of adiposity on depression [5], anxiety [6] and reduced quality of life [7]. However, there are inconsistencies in the literature, with several reviews reporting studies demonstrating no association between adiposity and psychological health [5], [6], [8]. In the extreme obese current understanding is limited as studies have focused on those individuals specifically seeking bariatric surgery [9], and have not included a range of assessments of putative psychological co-morbidities. In order to improve service provision for patients with extreme obesity, it is important to understand the extent of psychological co-morbidity and the impact on quality of life. Baseline data from treatment-seeking individuals at a community-based UK specialist weight management service were analysed as part of the service evaluation to examine the association between adiposity and psychological health.

## Materials and Methods

### Participants

This cross-sectional service evaluation study included a consecutive sample of 262 individuals with extreme obesity entering a specialist community-based weight management service (CWMS) in the West Midlands, UK. Eligible participants were referred to the CWMS by their general practitioner (GP) as a result of fulfilling the criteria of having a body mass index (BMI) ≥40 kg/m^2^, or alternatively a BMI ≥35 kg/m^2^ with a co-morbid health condition, such as type 2 diabetes mellitus or hypertension. Patient referral required previous unsuccessful weight loss attempts in primary care and commercial weight loss programmes. The participants entered the service between February 2008 and August 2012, with those included in the evaluation selected from an opportunity sample. Baseline data including adiposity, quality of life, and mental well-being were routinely collected prior to the initiation of treatment.

### Demographic information

As part of routine clinical care, demographic and health details were collected including participants’ age, gender, ethnicity, waist circumference, smoking status, and alcohol consumption. Details of participants’ co-morbid health conditions including cardiovascular disease, hypertension, diabetes, obstructive sleep apnoea and arthritis were also recorded.

### Clinical assessment

Within the CWMS, all participants underwent a comprehensive clinical history and examination by a consultant physician and specialist dietitian. A psychologist provided further support in the service. Participants’ initial weight and height data were recorded at baseline, and BMI was calculated by dividing participants’ weight in kg by height in meters squared.

### Quality of life and mental health measures

Questionnaires were routinely collected as part of the comprehensive clinical assessment. Quality of life and mental health were assessed using three measures, the Impact of Weight on Quality of Life (IWQOL-Lite) questionnaire, which is an obesity-specific quality of life measure, the EQ5D-3L, which is a general quality of life measure, and the Hospital Anxiety and Depression Scale (HADS) which is a screening tool widely used in both clinical and research settings. The IWQOL-Lite consists of 31 items measuring the impact of obesity on physical function, self-esteem, sexual life, public distress and work [10]. Respondents are asked to rate the extent to which a series of statements is applicable to them using a Likert scale ranging from 5 ‘Always true’ to 1 ‘Never true’. Responses to the questionnaire items yield a total impact of weight on quality of life score as well as individual scores for each of the five domains, with maximum scores of 100 on each subscale indicating optimum quality of life.

The EQ5D-3L consists of five questions relating to five dimensions of health; ‘mobility’, ‘self care’, ‘usual activities’, ‘pain and discomfort’ and ‘anxiety and depression’ [11]. Respondents indicate which of a possible three statements best describe their current health state for each dimension. A ‘level 1’ response indicates that the respondent has no problem in the specific dimension, a ‘level 2’ response indicates some problems, and a ‘level 3’ response indicates extreme problems. Respondents are asked to repeat this process for the five dimensions by indicating one level for each dimension, giving rise to scores ranging from 1 to 3, with scores of 3 on each dimension indicating the most severe impairment. Binary variables were computed for each dimension dichotomising the levels into ‘No problems’ (level 1) and ‘Problems’ (levels 2 and 3). Perceived current health state is measured by asking respondents to indicate their current health state on a Visual Analogue Scale (VAS) with endpoints labelled 0 ‘Worst imaginable health state’ and 100 ‘Best imaginable health state’.

The Hospital Anxiety and Depression Scale (HADS) comprises 14 items, 7 relating to anxiety, and 7 relating to depression [12]. Respondents rate the extent to which a series of statements represents how they currently feel using a Likert scale ranging from 0 to 3. The scale yields individual anxiety and depression scores as well as an overall HADS score. Individual subscale for anxiety and depressive symptom scores range from 0 to 21, with a score of 8 established as a cut-point for identifying symptoms of anxiety and depressive disorders and scores of 11 established as a cut-point for identifying more severe symptoms [13]. Whilst the HADS scale is widely used in clinical practice to identify the experience of symptoms of anxiety and depression, it cannot provide a confirmatory diagnosis of anxiety and depressive disorders.

### Statistical analysis

All data were analysed using SPSS (version 19.0). T-tests, cross-tabulation, chi^2^ and analysis of variance (ANOVA) calculations were conducted to compare the BMI tertile groups: first BMI tertile ≤42.99; second BMI tertile 43.00 − 48.61; third BMI tertile ≥48.62 kg/m^2^. Linear regression coefficients were calculated to assess the relationship between BMI and quality of life as measured by IWQOL-Lite scores (continuous) and in separate analyses between BMI and overall perceived health status as measured by EQ5D-3L VAS. Analyses were conducted for the whole sample and repeated in gender-stratified sub-groups.

Logistic regression models were constructed to assess the association between BMI and experience of symptoms of anxiety and depressive disorder as measured by HADS, and presence of problems in mobility, self care and performing usual activities as measured by EQ5D-3L, with the first BMI tertile group as the reference. The odds ratios (ORs) and 95% confidence intervals (CIs) for three hierarchical models are presented: crude; adjusting for age and sex; and additionally adjusting for the co-morbidities; diabetes, hypertension, arthritis, obstructive sleep apnoea and cardiovascular disease.

### Ethics statement

This was a retrospective analysis of routinely collected data to evaluate the psychological and quality of life burden of extreme obesity. Data were anonymised prior to any data analysis. The anonymised data were analysed as part of the specialist weight management service evaluation at the Heart of England NHS Foundation Trust, requiring no specific research ethics approval as recommended by the UK National Research Ethics Service [14]. The analysis was registered with the local governance audit department.

## Results

### Demographic and clinical characteristics

The participants were aged 19 to 76 years, with a mean age of 43.1±11.8 years and a mean BMI of 47.0±7.9 kg/m^2^. Table 1 shows that in those with increasing levels of adiposity, there were significantly more problems in mobility, self care and performing usual activities, and weight was reported to have a greater impact on physical function, causing public distress, ability to work, and overall quality of life. Those with increasing BMI were also more likely to have type 2 diabetes mellitus, hypertension, obstructive sleep apnoea (OSA) and cardiovascular disease (CVD). There were no significant differences in HADS anxiety and depression scores, with prevalence of anxiety and depressive symptoms consistently high across the BMI groups, with data for the combined sample indicating prevalence rates of anxiety symptoms (70.3%) and depressive symptoms (66.2%), which are far greater than the UK general population rates of 33.0% for anxiety disorders and 11.4% for depressive disorders [15]. Indeed, levels of severe anxiety and depressive symptoms defined by the higher cut-point scores of ≥11 are also substantial, with severe anxiety symptoms experienced by 48.3% of the sample and 40.4% of the sample experiencing symptoms of severe depressive disorders.

**Table 1:** Demographic and clinical characteristics of the sample across BMI tertiles.

	Whole sample	1^st^ BMI tertile ≤ 42.99	2^nd^ BMI tertile 43.00 − 48.61	3^rd^ BMI tertile ≥ 48.62	P
N	262	87	87	88	
Age (years)	43.1±11.8	43.0±13.2	41.7±10.2	44.7±11.8	0.234
Sex (% female)	74.8	72.4	77.0	75.0	0.783
**Ethnicity (%)**					0.585
White European	90.8	90.0	95.5	87.9	
Asian	5.6	5.0	2.3	8.6	
Black African/Caribbean	2.8	2.5	2.3	3.4	
Other	0.7	2.5	0.0	00	
Weight (kg)	132.1±24.7	112.6±14.7	129.1±15.6	154.2±22.3	<0.001
Body mass index (BMI, kg/m^2^)	47.0±7.9	39.4±2.6	45.8±1.6	55.8±6.6	<0.001
Waist circumference (cm)	131.6±14.5	123.8±11.8	128.6±12.7	142.4±12.5	<0.001
Systolic blood pressure (mmHg)	140.9±17.7	136.0±14.5	140.4±17.0	146.4±20.0	0.009
Diastolic blood pressure (mmHg)	85.2±11.7	83.3±9.7	85.5±11.4	86.9±13.6	0.263
Diabetes (%)	26.3	24.1	18.4	36.4	0.022
Hypertension (%)	34.4	31.0	26.4	45.5	0.022
Arthritis (%)	24.0	21.8	19.5	30.7	0.190
Obstructive sleep apnoea (OSA, %)	25.6	16.1	26.4	34.1	0.024
Cardiovascular disease (CVD, %)	11.1	9.2	5.7	18.2	0.026
Smoking (%)	25.5	31.7	23.0	22.2	0.655
Alcohol consumption (%)	64.7	62.9	81.0	52.9	0.004
**IWQOL-Lite (%)** Total	39.5±22.1	49.0±23.3	38.5±19.9	32.1±20.2	<0.001
Physical function	42.4±25.3	54.1±24.9	40.4±23.6	34.1±23.7	<0.001
Self-esteem	26.2±27.5	30.4±30.5	24.1±24.4	24.5±27.5	0.286
Sexual life	41.9±35.9	47.3±36.9	43.5±35.6	35.2±34.7	0.128
Public distress	40.5±28.9	56.6±31.5	38.9±25.2	27.7±22.7	<0.001
Work	51.2±30.3	61.6±30.4	46.1±27.4	47.1±31.3	0.003
**EQ5D**					
Mobility problems %	66.7	55.9	63.0	80.3	0.006
Self care problems %	35.7	29.9	27.2	50.0	0.006
Anxiety/depression problems %	75.3	73.1	75.6	77.0	0.864
Pain/discomfort problems %	85.3	80.0	86.3	89.3	0.272
Usual activities problems %	69.0	58.0	70.4	77.6	0.036
Perceived health status	44.0±20.1	47.4±19.4	42.4±19.0	42.9±21.8	0.303
**HADS** Total score	19.6±7.7	19.0±7.6	20.2±7.8	19.6±7.9	0.654
HADS anxiety score	10.4±4.5	10.5±4.3	10.7±4.8	10.2±4.5	0.780
HADS depression score	9.1±4.0	8.6±4.0	9.3±3.9	9.3±4.1	0.436
Anxiety symptoms ≥8 (%)	70.3	74.0	68.7	68.8	0.716
Depression symptoms ≥8 (%)	66.2	62.3	72.5	63.2	0.333
Severe anxiety symptoms ≥11 (%)	48.3	50.7	48.2	46.3	0.860
Severe depression symptoms ≥11 (%)	40.4	33.3	42.5	44.7	0.338

Data are percentages and means ± standard deviations. HADS = Hospital anxiety and depression scale.

Data indicate that quality of life is impaired, with sample mean IWQOL-Lite scores ranging from 26.2 (self-esteem) to 51.2 (work), whereby 100 represents optimum quality of life. Perceived health status was also poor with a sample mean of 44.0, whereby 100 represents best possible health state; which is considerably worse than the UK general population mean score of 82.8 [16]. There were no significant gender differences in EQ5D-3L. However there were gender differences in HADS anxiety, but not depression and total scores, and gender differences in the IWQOL-Lite total and subscales self-esteem and sexual life, with significantly poorer quality of life in females (data not shown).

### Linear regression: BMI, IWQOL-Lite and perceived health status

The IWQOL-Lite total measure and the subscales physical function, self-esteem, public distress and work were significantly negatively associated with increasing BMI (Table 2). Increasing BMI was associated with decreasing quality of life across the domains of physical function (1.93, p<0.001), self-esteem (1.62, p<0.05), public distress (2.69, p<0.001), work (1.33, p<0.05) and total score (1.79, p<0.001). Stratification by gender revealed that BMI was more strongly negatively associated with these measures in males. Interestingly, BMI was not significantly associated with the sexual life IWQOL-Lite subscale and the perceived health status measure (EQ5D-3L VAS).

**Table 2:** Linear regression of BMI predicting IWQOL-Lite subscale and total scores and perceived health status (EQ5D-3L VAS) in whole sample and stratified by gender.

	Univariate			Model 1			Model 2		
	U.B.	S.E.	S.B	U.B.	S.E.	S.B	U.B.	S.E.	S.B
**Physical function**	−0.83**	0.20	−0.26	−1.95**	0.48	−0.62	−1.93**	0.47	−0.61
Male †	−1.66*	0.54	−0.37	−1.92*	0.53	−0.43	−2.00**	0.50	−0.44
Female †	−0.66*	0.21	−0.23	−0.56*	0.20	−0.19	−0.51*	0.21	−0.18
**Self esteem**	−0.34	0.22	−0.10	−1.53*	0.53	−0.44	−1.62*	0.53	−0.47
Male †	−1.80*	0.63	−0.35	−1.39*	0.60	−0.27	−1.19	0.63	−0.23
Female †	0.01	0.22	−0.00	−0.06	0.22	−0.02	−0.11	0.22	−0.04
**Sexual life**	−0.56	0.32	−0.12	−1.55*	0.77	−0.34	−1.45	0.79	−0.31
Male †	−1.47	0.75	−0.25	−1.55*	0.76	−0.27	−1.21	0.81	−0.21
Female †	−0.33	0.35	−0.08	−0.27	0.35	−0.06	−0.29	0.36	−0.07
**Public distress**	−1.44**	0.22	−0.40	−2.82**	0.53	−0.77	−2.69**	0.54	−0.74
Male †	−3.00**	0.49	−0.62	−2.76**	0.48	−0.57	−2.48**	0.51	−0.51
Female †	−1.14**	0.24	−0.34	−1.19**	0.24	−0.35	−1.18**	0.25	−0.35
**Work**	−0.83*	0.25	−0.22	−1.34*	0.65	−0.35	−1.33*	0.66	−0.35
Male †	−1.39*	0.67	−0.28	−1.23	0.68	−0.25	−1.22	0.73	−0.25
Female †	−0.70*	0.27	−0.20	−0.70*	0.28	−0.20	−0.68*	0.28	−0.19
**IWQOL−Lite total**	−0.79**	0.17	−0.28	−1.84**	0.43	−0.66	−1.79**	0.44	−0.65
Male †	−1.82**	0.44	−0.47	−1.78**	0.45	−0.46	−1.60*	0.46	−0.42
Female †	−0.56*	0.19	−0.22	−0.54*	0.19	−0.22	−0.52*	0.19	−0.21
**Perceived health status**	−0.17	0.18	−0.07	−0.12	0.49	−0.05	−0.07	0.49	−0.03
Male †	−0.12	0.48	−0.04	−0.11	0.50	−0.03	−0.08	0.50	−0.03
Female †	−0.18	0.19	−0.07	−0.18	0.20	−0.07	−0.12	0.20	−0.05

U.B.  = Unstandardized Beta, S.E.  = Standard error, S.B  = Standardised Beta.

*P<0.05, **P<0.001.

Model 1 adjusting for age, gender and interaction between BMI and gender.

† Model 1 and 2 adjusting for age only.

Model 2 additionally adjusting for diabetes, hypertension, arthritis, obstructive sleep apnoea, cardiovascular disease.

### Logistic regression: BMI, EQ5D-3L and HADS anxiety and depression

Table 3 shows the logistic regression analyses of the EQ5D-3L subscales ‘mobility’, ‘self care’ and ‘usual activities’, which were significantly associated with BMI. The fully adjusted model revealed an increased risk of mobility problems with increased BMI, with the odds ratios of 1.64 (0.78 − 3.44) and 3.44 (1.47 − 8.05) for second and third BMI tertile groups (P for trend <0.05) respectively, compared to those in the first BMI tertile group. There was a non-significant increased risk of self care problems, with the odds ratios of 0.89 (0.41 − 1.96) and 1.87 (0.86 − 4.09) for second and third BMI tertile groups (P for trend  = 0.104) respectively. However the fully adjusted model remained significant demonstrating increased risk of self care problems in the continuous BMI model 1.05 (1.00 − 1.09). The fully adjusted model also revealed an increased risk of problems performing usual activities, with the odds ratios of 2.04 (0.98 − 4.26) and 2.45 (1.10 − 5.46) for second and third BMI tertile groups (P for trend <0.05) respectively, compared to those in the first BMI tertile group. Interestingly, the logistic regression analyses of anxiety and depressive symptoms as defined as HADS subscale score ≥8 revealed that anxiety and depressive symptoms were not significantly associated with BMI across the range encountered in the sample.

**Table 3:** Logistic regression of presence of problems in mobility, self care and usual activities (EQ5D) and presence of anxiety and depression (HADS) by BMI tertiles and by continuous BMI.

		1^st^ tertile ( ≤ 42.99)	2^nd^ tertile (43.00 − 48.61)	3^rd^ tertile ( ≥ 48.62)	P for linear trend	Continuous BMI
		Odds ratio (95% CI)				
**Mobility problems**	**Univariate**	1.00	1.25 (0.64 − 2.44)	2.95 (1.40 − 6.22)*	0.004	1.07 (1.02 – 1.12)*
	**Model 1**	1.00	1.32 (0.66 – 2.63)	2.83 (1.31 – 6.15)*	0.008	1.07 (1.02 – 1.12)*
	**Model 2**	1.00	1.64 (0.78 – 3.44)	3.44 (1.47 – 8.05)*	0.009	1.08 (1.03 – 1.13)*
**Self care problems**	**Univariate**	1.00	0.84 (0.41 – 1.72)	2.19 (1.09 – 4.39)*	0.018	1.05 (1.01 – 1.09)*
	**Model 1**	1.00	0.88 (0.42 – 1.88)	2.05 (0.98 – 4.27)	0.044	1.05 (1.01 – 1.10)*
	**Model 2**	1.00	0.89 (0.41 – 1.96)	1.87 (0.86 – 4.09)	0.104	1.05 (1.00 – 1.09)*
**Problems performing usual activities**	**Univariate**	1.00	1.71 (0.86 – 3.37)	2.65 (1.27 – 5.52)*	0.008	1.05 (1.01 – 1.10)*
	**Model 1**	1.00	1.73 (0.86 – 3.44)	2.48 (1.17 – 5.23)*	0.016	1.05 (1.01 – 1.10)*
	**Model 2**	1.00	2.04 (0.98 – 4.26)	2.45 (1.10 – 5.46)*	0.040	1.05 (1.00 – 1.10)*
**Anxiety**	**Univariate**	1.00	0.75 (0.37 – 1.51)	0.73 (0.36 – 1.45)	0.409	0.98 (0.95 – 1.02)
	**Model 1**	1.00	0.68 (0.33 – 1.42)	0.74 (0.35 – 1.54)	0.460	0.98 (0.94 – 1.01)
	**Model 2**	1.00	0.71 (0.34 – 1.50)	0.81 (0.37 – 1.74)	0.633	0.98 (0.95 – 1.02)
**Depression**	**Univariate**	1.00	1.57 (0.78 – 3.15)	1.00 (0.51 – 1.98)	0.958	1.00 (0.96 – 1.03)
	**Model 1**	1.00	1.59 (0.79 – 3.20)	1.06 (0.53 – 2.11)	0.910	1.00 (0.96 – 1.03)
	**Model 2**	1.00	1.65 (0.80 – 3.40)	1.26 (0.61 – 2.62)	0.550	1.00 (0.97 – 1.04)

Presence of problems defined as level 2 (some problems) and level 3 (extreme problems) scores on EQ5D-3L; Presence of anxiety and depression defined as HADS anxiety subscale score ≥8.

* P<0.05.

**P<0.001.

Model 1 adjusting for age and sex.

Model 2 additionally adjusting for diabetes, hypertension, arthritis, obstructive sleep apnoea, cardiovascular disease.

## Discussion

The findings of the present evaluation demonstrated that quality of life was markedly impaired in this sample of extreme obese individuals entering a specialist community-based weight management service having not succeeded with previous efforts at weight loss. Furthermore, we observed that the prevalence of anxiety and depressive symptoms was very high. Whilst much of the research investigating the complex association between adiposity and quality of life and mental well-being has incorporated individuals across the spectrum of obesity, the present study is of particular importance as it focuses on the escalating extreme obese population [4].

We observed a significant negative association between increasing adiposity at these extreme levels and quality of life, specifically in the areas of physical function, self-esteem, public distress and work, with increased adiposity associated with reduced quality of life as measured by IWQOL-Lite score. The association between adiposity and weight-specific quality of life as measured by IWQOL-Lite has been established, with findings indicating that BMI accounts for approximately 28% of the variance in total IWQOL-Lite scores [7]. Previous research has shown that scores vary with degree of adiposity and treatment status, with those with higher BMI and those seeking treatment reporting significantly worse quality of life [7]. In addition, changes in IWQOL-Lite score from baseline to post-intervention have been shown to correlate significantly with weight loss [17]. The results obtained in the present study show a similar level of impairment in quality of life to those obtained in a study of bariatric surgery-seeking individuals, which reported scores ranging from 40.4 (work) to 46.2 (self-esteem) across IWQOL-Lite subscales [7]. Interestingly, the present study reported one subscale which was not shown to be significantly associated with adiposity; sexual life. This is consistent with findings from a comparison of white and African American US women, whereby white women scored significantly lower on sexual life compared to their African American counterparts, in both class II and III obesity, and BMI was significantly associated with sexual life in the white sub-group but not in the African American sub-group [18]. A similar pattern of results whereby significant association was not observed between BMI and sexual life has also been reported in a sample of over 400 bariatric surgery-seeking extreme obese individuals [18]. The investigators concluded that the lack of observed association is due to the high level of co-morbidities, which may diminish the association between quality of life and BMI at the level of extreme obesity. However, the results of the present study show that the association between BMI and quality of life remains when controlling for co-morbid health conditions, suggesting that obesity negatively affects quality of life, independently of these conditions. This lack of association indicates that there are additional factors outside of those measured, which contribute to the reduced level of quality of life in these specific domains. Indeed, the substantial impairments in sexual quality of life in this population have been greatly under-researched [19] and are thought to be associated with the broader aspects of stigmatisation and discrimination [20] as well as negative perceived body image [19], [21] experienced by this patient population.

Likewise, significant associations were observed between adiposity and some, but not all, of the EQ5D-3L subscales. Adiposity was associated with experience of problems in mobility, self-care, and performing usual activities, with those in the third BMI tertile more likely to experience problems in these areas. Whilst the fully adjusted models remained significant for the mobility and performing usual activities analyses, the self-care model was no longer significant when fully adjusted in the BMI tertile model. These findings are consistent with the limited previous studies which have shown that general quality of life as measured by EQ5D-3L is poorest for individuals with class III obesity, compared to class I and II obese groups, as well as overweight and underweight groups, relative to those of normal weight where quality of life scores are optimum [22],[23]. Notably, the present study reported two subscale scores, which were not shown to be significantly associated with adiposity, ‘pain and discomfort’ and ‘anxiety and depression’(data shown in Table 1). This is in contrast to previous research which has identified that obese individuals are at greater odds of experiencing anxiety and depressive disorders [5],[6], and pain (OR = 1.94) relative to normal weight counterparts [23]. The results of the present study indicate that individuals reported the greatest amount of problems in these domains. Likewise, no significant association between adiposity and perceived health status (EQ5D-3L VAS) was observed. Together, these findings suggest a possible ceiling effect for the EQ5D-3L tool being unable to detect differences within such a homogeneous group as the present sample in which the quality of life is consistently low. An additional aspect is the absence of normal weight individuals, which truncates the BMI range, reducing the opportunity to identify an association. There was a non-significant trend whereby perceived health status was highest for the first BMI tertile group (47.4) and lowest for the third BMI tertile group (42.9) supporting the above contentions. Unlike the present study, previous studies have demonstrated that perceived health status significantly decreases with increasing adiposity, and is poorest for individuals with class III obesity, relative to those of normal weight [23]; it is likely that the expected significant negative association was not observed in the present study due to the homogeneity in adiposity of this exclusively extreme obese sample.

The expected significant associations between adiposity and symptoms of anxiety and depressive disorder as measured by HADS were also not observed in the present study. However, it was evident that the prevalence of symptoms of psychological co-morbidities was high across the sample (anxiety, 70.3%; depression, 66.2%) and is far greater than the UK general population rates of 33.0% for anxiety and 11.4% for depressive disorder [15]. The relationship between depressive disorders and obesity has been widely documented in the literature, with results from prospective studies indicating that obesity is associated with future incidence of depression and cross-sectional studies revealing significant positive associations between adiposity and depression, particularly in females [5]. No significant variation in prevalence of anxiety and depressive symptoms was observed across the levels of adiposity in the current sample, probably due to the truncated BMI range and the overall high prevalence of anxiety and depressive symptoms. However, data from the NHANES (National Health and Nutrition Examination Survey) study demonstrated a dose-response relationship between depression and adiposity, with class III obese individuals having greater odds of experiencing lifetime major depression (OR = 2.60), recurrent major depression (OR = 2.28), depression in the past month (OR = 4.98) and past year (OR = 2.92) than the class I and II obesity groups relative to those of normal BMI [24].

Whilst much research has demonstrated evidence for an association between adiposity and both depressive and anxiety disorders [6], [24], [25] it is important to note that some studies have reported no significant association [8] or have reported non-significant trends [26]–[29]. A systematic review and meta-analysis of the association between obesity and anxiety has concluded that there is evidence in support of a positive association between obesity and anxiety, with pooled cross-sectional data indicating that obese individuals are more likely to experience anxiety (OR = 1.4) [6]. It is also of interest that the findings of the present work indicate that anxiety was more prevalent in this sample than depression.

The mechanism of the association between adiposity, impairment in quality of life and presence of anxiety and depressive disorders is not yet established, with several proposed pathways through which obesity may lead to psychological co-morbidity and vice versa. Firstly, through the multiple health threats associated with obesity acting as stressors, and secondly through the negative effects of stigma and weight-related discrimination. Indeed frequency of stigmatisation and inability to adopt effective coping strategies have been shown to result in depressed mood [29]. The relationship between obesity and depression specifically, appears to be bi-directional with obesity associated with increased experience of depressive symptoms, and depressive episodes associated with further weight gain. Furthermore, obese individuals are more likely to over-eat and gain weight compared to non-obese individuals during an episode of depression [30]. A systematic review of the relationship between depression and adiposity reported that the majority of evidence was cross-sectional and thus causality could not be established [5].

Previous research has been criticised for the inclusion of only one measure of quality of life [23], and as such the inclusion of several quality of life measures is a novel aspect of the present evaluation. Previous studies have utilised either general measures such as the EQ5D-3L [22],[23] and the Medical Outcomes Study Short Form Health Survey (SF-36) [31],[32] or condition specific measures such as the Obesity Adjustment Survey (OAS) [33] and the Obesity Related Well-being (ORWELL 97) questionnaire [34]; however the IWQOL-Lite measure is the most widely used weight-specific measure [35]. Present findings suggest that in the assessment of quality of life within extreme obesity, both weight-specific and general quality of life measures are effective. Notably, the IWQOL-Lite, EQ5D-3L and HADS measures all contained subscales which were not associated with adiposity indicating that each of the tools may have limitations in capability of identifying differences in extreme adiposity. The specific domains which were not associated with adiposity; pain and discomfort, anxiety and depression and sexual life were in fact the more severely affected aspects of life. Likewise, none of the components of the HADS mental health screening tool were associated with adiposity. However, the tool was shown to be effective in determining prevalence of symptoms of anxiety and depressive disorders in an extreme obese sample. Future studies should utilise several quality of life measures, including those that are weight specific and general in order to establish the validity of the measures in this patient group [36], as well as enabling deeper understanding of the additional factors which may influence the complex relationship between adiposity, quality of life and mental well-being.

A key strength of the present study is that it demonstrates that the negative impact on quality of life associated with increasing BMI remains even when controlling for the presence of obesity related co-morbid health conditions commonly experienced by the extreme obese population. Previous studies have concluded that the association between adiposity and quality of life is mediated by health co-morbidities such as pain, cardiovascular disease and type 2 diabetes mellitus [37]–[39]; however the present study supports that the association is independent, and that the role of co-morbidities in the relationship may have previously been over-estimated, and the impact of adiposity, underestimated.

An additional strength of the present study is that it improves understanding of the characteristics of this less-researched extreme obese population. However, the present work has several limitations. The individuals characterised in the sample are those that had sought assistance in managing their weight and as a consequence it may not be appropriate to extrapolate these findings to the general extreme obese population, as evidence suggests that non-treatment-seeking obese individuals do not experience the same psychological co-morbidities [40] and impairment in quality of life [7] as those seeking treatment. Furthermore, the fact that these findings are obtained from a single weight management service may limit their generalizability to other UK specialist weight management settings. The cross-sectional design of the present study means that it is not possible to confirm a causal relationship between adiposity and quality of life. The present work also adopted a service evaluation approach aiming to understand the psychological and quality of life burden of obesity to improve service provision, which means that it is not possible to compare the characteristics of the extreme obese treatment-seeking group, with a non-obese control group or an extreme obese non-treatment-seeking group for comparison. As such the findings of the present work should be interpreted with caution and future studies adopting a controlled design comprising a control group of extreme obese non-treatment-seeking individuals should be utilised in further investigation of the psychological co-morbidities of extreme obesity.

In conclusion, we observed that among this sample of treatment-seeking extreme obese individuals there is a high prevalence of symptoms of psychological co-morbidity, including experience of anxiety and depressive disorder symptoms and reduced quality of life. Increasing adiposity was associated with a reduction in several areas of quality of life, but was not significantly associated with symptoms of anxiety and depressive disorders. These findings are of clinical importance indicating that the impairment in quality of life and mental health challenges faced by these individuals must be addressed and incorporated into the multi-disciplinary care of these patients, in order to provide tailored weight management interventions.
